# Transforming into a Learning Health System: A Quality Improvement Initiative

**DOI:** 10.1097/pq9.0000000000000724

**Published:** 2024-05-09

**Authors:** Jennifer L. Chiem, Elizabeth E. Hansen, Nicolas Fernandez, Paul A. Merguerian, Sanjay R. Parikh, Kayla Reece, Daniel K. Low, Lynn D. Martin

**Affiliations:** * From the Department of Anesthesiology and Pain Medicine, Seattle Children’s Hospital, Seattle, Wash.; † Department of Anesthesiology and Pain Medicine, University of Washington, Seattle, Wash.; ‡ Department of Urology, Seattle Children’s Hospital, Seattle, Wash.; § Department of Urology, University of Washington, Seattle, Wash.; ¶ Seattle Children’s Hospital, Seattle, Wash.; ∥ Department of Otolaryngology—Head and Neck Surgery, University of Washington, Seattle, Wash.; ** Department of Perioperative Services, Seattle Children’s Hospital, Seattle, Wash.

## Abstract

**Background:**

The Institute of Medicine introduced the Learning Healthcare System concept in 2006. The system emphasizes quality, safety, and value to improve patient outcomes. The Bellevue Clinic and Surgical Center is an ambulatory surgical center that embraces continuous quality improvement to provide exceptional patient-centered care to the pediatric surgical population.

**Methods:**

We used statistical process control charts to study the hospital’s electronic health record data. Over the past 7 years, we have focused on the following areas: efficiency (surgical block time use), effectiveness (providing adequate analgesia after transitioning to an opioid-sparing protocol), efficacy (creating a pediatric enhanced recovery program), equity (evaluating for racial disparities in surgical readmission rates), and finally, environmental safety (tracking and reducing our facility’s greenhouse gas emissions from inhaled anesthetics).

**Results:**

We have seen improvement in urology surgery efficiency, resulting in a 37% increase in monthly surgical volume, continued adaptation to our opioid-sparing protocol to further reduce postanesthesia care unit opioid administration for tonsillectomy and adenoidectomy cases, successful implementation of an enhanced recovery program, continued work to ensure equitable healthcare for our patients, and more than 85% reduction in our facility’s greenhouse gas emissions from inhaled anesthetics.

**Conclusions:**

The Bellevue Clinic and Surgical Center facility is a living example of a learning health system, which has evolved over the years through continued patient-centered QI work. Our areas of emphasis, including efficiency, effectiveness, efficacy, equity, and environmental safety, will continue to impact the community we serve positively.

## INTRODUCTION

In 2006, the Institute of Medicine, known as the National Academy of Medicine, introduced the Learning Healthcare System (LHS) concept “to apply the best evidence for the collaborative healthcare choices of each patient and provider and to ensure innovation, quality, safety, and value in health care.”^1^ The LHS paradigm aligns science, informatics, incentives, and culture to allow continuous improvement and innovation. Integrating new technology and research improves patient care and outcomes.^2^

### Problem Description

Healthcare advancement is historically slow. As modern medicine develops newer treatments, we should adapt our practice. Core elements for an LHS include: “(1) data infrastructure, including the collection and reuse of electronic health record data; (2) care improvement targets that assist learning and health through clinical decision-making activities; and (3) a supportive policy environment in which financial incentives reward high-value care and promote performance transparency.”^3,4^

### Available Knowledge

The United States has several macro LHS examples. Vanderbilt University Medical Center established an LHS platform to create generalizable knowledge.^5,6^ Additionally, the Veterans Affairs Evidence Synthesis Program supports the Veterans Health Administration healthcare system goals using the US Department of Veterans Affairs Quality Enhancement Research Initiative Implementation Roadmap.^7,8^ Third, John Hopkins Community Physicians have implemented initiatives in the primary care setting based on LHS elements.^9^ There are limited published pediatric center LHS examples.^10–12^

### Rationale

The Bellevue Clinic and Surgery Center (BCSC) is an ambulatory outpatient facility formally affiliated with Seattle Children’s Hospital, where clinicians incorporate standardized clinical pathways into their practice. The ambulatory surgical center (ASC) leadership team embraced lean methodology and the LHS concept upon opening in 2010. Over the years, it has transformed into a micro LHS in clinical practice.

### Specific Aims

Over the last 7 years, we integrated LHS practice into a pediatric ASC to improve efficiency, effectiveness, efficacy, equity, and environmental safety.

## METHODS

### Context

BCSC is an ASC for a healthy patient population presenting for low-acuity surgery. Surgical subspecialties include otolaryngology, urology, general surgery, ophthalmology, plastic surgery, oral surgery, and orthopedic surgery. Common surgical procedures include myringotomy tubes, adenotonsillectomy, buried penis repair, inguinal and umbilical hernia repair, strabismus surgery, nevus removal, shoulder and knee arthroscopy, and anterior cruciate ligament reconstruction. Our anesthesiology group includes 12 pediatric anesthesiologists and 23 certified registered nurse anesthetists (CRNA). The perioperative staff developed standardized clinical protocols centered on evidence-based practice to improve the quality and consistency of clinical care. This structure, coupled with a data-driven culture enabled by new information technology software in 2015 and a steady commitment to improve and innovate, has created an ideal environment for quality improvement (QI) work. The protocols undergo frequent and concurrent QI cycles based on data results. The medical staff agreed to transparency regarding available performance measures. Monthly anesthesiology staff meetings have dedicated time to discuss process control chart data from current QI work and to brainstorm future ideas. Daily staff morning huddle includes a discussion of current QI initiatives. Joint staff meetings with nurses provide an opportunity for collaborative projects. The electronic anesthesiology record has prompts to remind providers of protocol changes. Process control charts are visible in the anesthesia workroom and nursing bulletin boards. Our institution is well equipped to track the implementation and effects of multiple simultaneous practice changes due to the informatics infrastructure built around electronic medical record (EMR) data.

### Measures

#### Analysis

AdaptX (Seattle, Wash.), a software program that allows clinicians to extract continuously updated, aggregated health information from the hospital’s EMR, was used to chart and analyze data using statistical process control methods.^13^

We used standard statistical process control rules to identify special cause variation (SCV). P-charts displayed postoperative opioid rescue administration in the postanesthesia care unit (PACU). X-bar charts displayed PACU maximum pain score, PACU length of stay (LOS), and postoperative prescription opioid doses. Every X-bar chart has a paired S chart. A T chart displays the 30-day readmission rate. We set control limits at three sigma above and below the mean.^14^

#### Ethical Considerations

This project was submitted to the Seattle Children’s institutional review board and was not deemed a research study. We did not require further review.

### Interventions

**Fig. 1.**
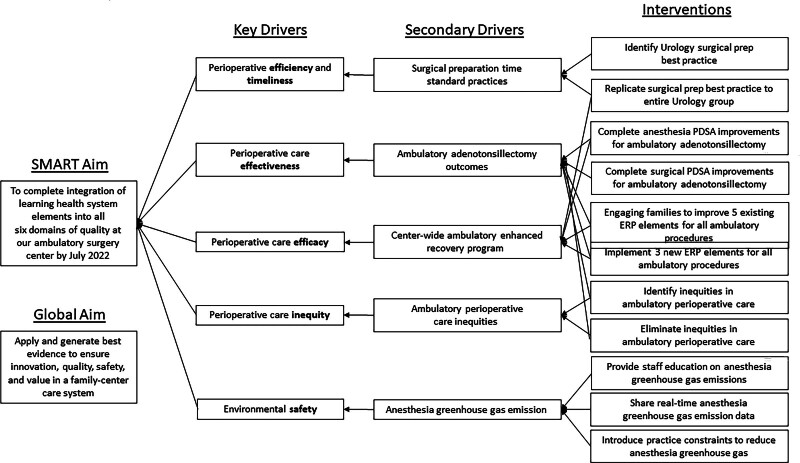
Key driver diagram.

Representative examples that address all six domains of quality while embracing LHS elements over the past 7 years include (1) efficiency regarding surgical block time use, (2) effectiveness in providing adequate analgesia after transitioning to an opioid-sparing anesthesia protocol, (3) efficacy in terms of creating a center-wide pediatric Enhanced Recovery Program (ERP), (4) equity by evaluating for racial disparities in surgical readmission rates, and lastly, (5) environmental safety, by tracking and reducing our facility’s greenhouse gas (GHG) emissions from inhaled anesthetics (Fig. 1), and (6) our family-centered care approach includes in-person interpreters available on the day of surgery, optional child life involvement preoperatively and on the day of surgery, and a multilingual cellular phone application providing perioperative instructions.

### Study of the Interventions/Results

#### Efficiency

Efficiency is avoiding wasted materials, time, effort, or money to complete a desired process. Ambulatory surgical services rely on operating room efficiency to meet patient demands. We standardized our pediatric urology practice for tasks performed between “Anesthesia Ready” and “Surgery Start Time” (surgical preparation time), aiming to increase our ambulatory operating room throughput. Shortening surgical preparation times adds the opportunity for more surgical cases each day. This improved efficiency reduces surgical case backlog, improving the timeliness of care for patients and families to schedule surgical dates.

A multidisciplinary group developed a plan to optimize surgical preparation time. The first Plan-Do-Study-Act (PDSA) cycle identified surgeons with the lowest surgical preparation times using EMR data from January 1, 2019, to August 30, 2020. Their surgical preparation time workflows were reviewed and discussed amongst the urology group. As a result, the team developed a clinical standard work protocol, implemented it on September 1, 2020, and studied it through September 1, 2021. The surgical preparation time analysis had a total of 2,506 patients. Of those, 1,333 underwent surgery before implementing the new clinical standard work protocol and 1,173 after implementation. The new protocol showed SCV with an average time improvement in surgical preparation time of 3 minutes and for all surgeons of 1.8 minutes. The total number of cases performed after protocol implementation increased from 70 to 96 cases per month at the end of the study period (37% increase). In the first month after implementation, the total cases increased to 99, and for the remaining study period, there was a median of 102 (SD 14) cases per month. The baseline “Last Case End Time” was 15.7 minutes later than the scheduled end time. After protocol implementation, it improved to 20.8 minutes before the scheduled end time. It is important to note that the staffing model remained the same—the surgical backlog reduction improved the timeliness of care for patients and families.

#### Effectiveness

**Fig. 2.**
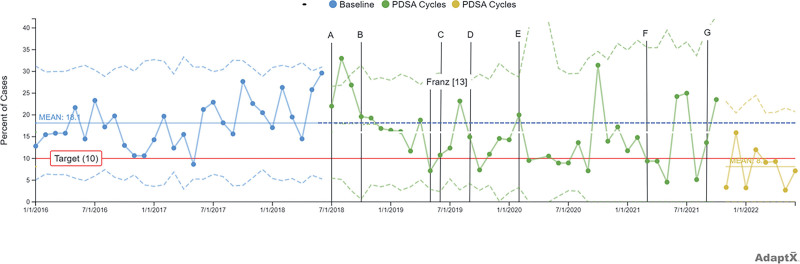
P-Chart: PACU rescue IV opioids rate; Baseline (morphine and acetaminophen), (A) dexmedetomidine and ibuprofen, (B) dexmedetomidine and ketorolac, (C) dexamethasone ×2, (D) no local, € IV lidocaine, (F) ketamine 0.2 mg/kg, (G) ketamine 0.4 mg/kg. Annotation of previously published QI work by Franz et al.^13^

The Oxford Dictionary defines effectiveness as “the degree to which something successfully produces a desired result.” We continuously use the LHS concept to improve the effectiveness of patient care delivery and outcomes. To illustrate this, we will review the care delivery process for ambulatory tonsillectomy and adenoidectomy (T&A) previously published.^13^ Originally, the surgical team improved outcome measures defined as PACU maximum pain scores, rescue opioid rate, and mean LOS. The outcome measures were all stable, whereas the rate of postoperative nausea and vomiting and cost of anesthetic medication per case decreased. A further example of the dynamic living use of LHS principles is the continued pursuit of improvement after the publication of this opioid-sparing anesthetic technique in 2019. Because these efforts, we completed six additional PDSA cycles of improvement (four anesthesia and two surgical). The primary goal of these efforts has remained better effectiveness. Specifically, the team is attempting to reduce the rate of postoperative opioid utilization (rescue morphine use in PACU: target <10% of cases) while decreasing care delivery costs. Five PDSA cycles (four anesthesia and one surgery) have focused on refining the analgesia protocols based on published best evidence. We accepted or rejected the interventions based on the observed clinical effectiveness. In order, the cycles evaluated were higher dose dexamethasone 0.3 mg/kg (rejected), no surgical lidocaine local infiltration (accepted), intravenous lidocaine 1.5 mg/kg (rejected), ketamine 0.2 mg/kg (rejected) and ketamine 0.4 mg/kg (accepted). The numerous PDSA cycles for our T&A protocol have yielded stable PACU maximum pain scores after PDSA cycle A (**Figure 1**, **Supplemental Digital Content 1,** which describes X-bar chart: PACU maximum pain score, http://links.lww.com/PQ9/A547; **Figure 2**, **Supplemental Digital Content 2,** which describes PACU maximum pain score S chart, http://links.lww.com/PQ9/A548) while achieving SCV for PACU opioid rescue rate reduction below the 10% target (Fig. 2). There is SCV present with the addition of ketamine at doses of 0.4 mg/kg. The primary balancing measure (PACU LOS) remained stable.

#### Efficacy

The Oxford Dictionary defines medical efficacy as “the ability of an intervention (drug or surgery) to produce the desired beneficial effect.” With the recent completion of an ERP, we used LHS principles to continuously improve the efficacy of patient care delivery in the entire ASC.^15^ We have included additional postoperative follow-up data.

**Fig. 3.**
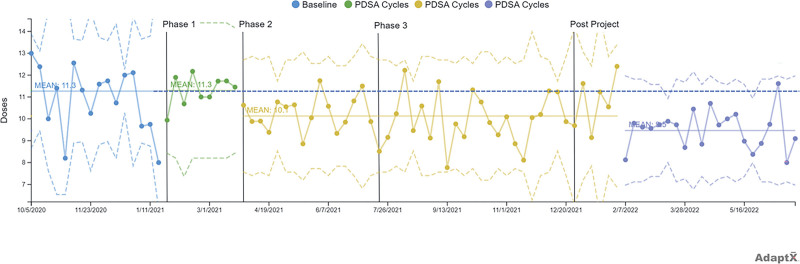
X-bar chart: average postoperative opioid prescription dose count.

**Fig. 4.**
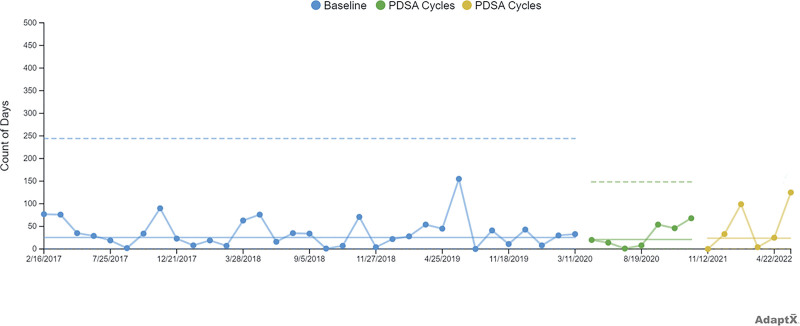
T chart: days between 30-day return to OR.

**Fig. 5.**
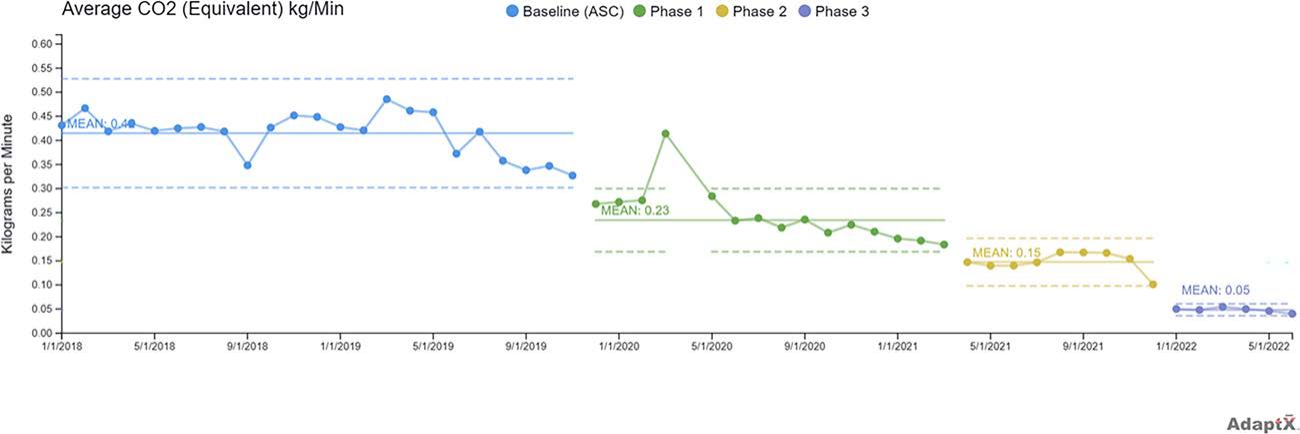
Average GHG emissions from anesthesia gases in kg CO_2_e/min, at baseline and through three intervention phases. Modified from the original article.^25^

**Fig. 6.**
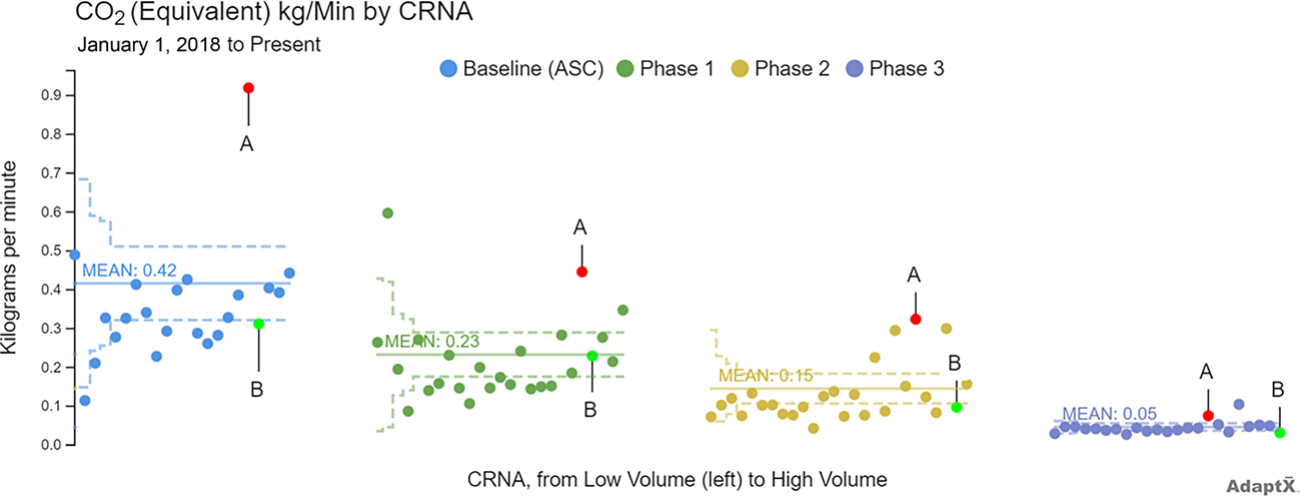
Funnel plot: average GHG emissions from anesthesia gases by CRNA, in kg CO_2_e/min, at baseline and through three phases of intervention, by the number of cases.

Utilizing LHS processes and behaviors, including enhanced patient and parent educational interactions both pre- and postoperatively, the team implemented five new pediatric-specific ERP elements while improving three existing ERP ones. We saw increases in PACU LOS, maximum PACU pain score, and PACU rescue opioid administration rate. Subsequently, the interdisciplinary team focused efforts on postoperative opioid prescriptions and pain. With the implementation of our ERP, the mean PACU LOS showed SCV in phase 1, which was sustained in phases 2 and 3 (**Figure 3, Supplemental Digital Content 3**, which describes X-bar chart: PACU LOS, http://links.lww.com/PQ9/A549; **Figure 4, Supplemental Digital Content 4**, which describes PACU LOS S chart, http://links.lww.com/PQ9/A550). In addition, the maximum PACU pain score also had SCV in phase 1, which was sustained throughout the project (**Figure 5, Supplemental Digital Content 5**, which describes X-bar chart: PACU maximum pain score, http://links.lww.com/PQ9/A551; **Figure 6, Supplemental Digital Content 6**, which describes PACU maximum pain score S chart, http://links.lww.com/PQ9/A552). There was increased use of rescue opioids in PACU in phase 1, which was sustained throughout the project (**Figure 7, Supplemental Digital Content 7**, which describes P-Chart: PACU rescue IV opioid rate, http://links.lww.com/PQ9/A553). During the COVID era, our ASC performed higher acuity cases that were not amenable to regional anesthesia with a planned transfer back to our main hospital, which accounts for our higher PACU LOS, pain scores, and opioid rescue rates. A major shortcoming of the initial ERP effort was limited follow-up information after discharge from the ASC. In subsequent postproject work, the surgical team completed several PDSA cycles to reduce the number of opioid doses prescribed to patients appropriately. As a result, there was a reduction across all surgical specialties in the number of opioid doses prescribed to patients with SCV (Fig. 3, **Figure 8, Supplemental Digital Content 8**, which describes average postoperative opioid prescription dose count S chart, http://links.lww.com/PQ9/A554). Furthermore, there was no increase in the rate of opioid prescription refills within 90 days of surgery.

#### Equity

There is literature to support healthcare disparities within anesthesiology.^16,17^ In one study, patients with lower socioeconomic status received fewer antiemetics.^16^ Another study determined that Latino patients received less analgesic medication than non-Latino patients.^18^ Green et al^19^ determined a consistent undertreatment of pain for African Americans and other minorities. We wanted to determine if racial disparities exist by examining 30-day readmission and reoperation rates for pediatric tonsillectomy with and without adenoidectomy cases.

We included the American Society of Anesthesiologists physical status 1–2 patients 2–18 years of age undergoing ambulatory tonsillectomy with and without adenoidectomy at BCSC from December 1, 2016, to June 30, 2022. We studied baseline data from December 1, 2016, to June 30, 2022. The team performed 2,950 cases. The mean 30-day reoperation rate was 1.1% of cases. We stratified the data utilizing funnel charts. Based on patient race and ethnicity, the percentage of cases requiring a readmission for families identified as Asian and Black or African American was 2.3%, Hispanic was 1.0%, compared with non-Hispanic White at 0.6%.

We presented the findings to hospital leadership and perioperative teams beginning on July 1, 2020. For PDSA cycle 1, two multidisciplinary team meetings in April and May 2021 brainstormed solutions to bridge the racial disparity gap for the patient population. The BCSC teams began their rapid process improvement project on September 1, 2021 to discharge families with a visual cue hydration chart indicating how many cups the patient needed to drink daily.

During PDSA cycle 1 (July 1, 2020, to June 30, 2022), the team performed 783 tonsillectomies with and without adenoidectomy cases. The mean 30-day reoperation rate was 1.7% of cases. Based on patient race and ethnicity, the percentage of cases requiring a readmission for families identified as Asian was 0% (low case numbers), Black or African American was 1.5%, Hispanic was 1.5%, compared with Non-Hispanic White at 1.6%. There was no SCV due to the rarity of admission. A T chart shows days between readmission events (Fig. 4).

#### Environmental Safety

Inhaled anesthetic gases are a significant source of GHG emissions from surgical care. At Seattle Children’s Hospital, emissions from anesthetic gasses comprised 5%–7% of total GHG emissions. This problem has been known for years, with a sustainability manager presenting data to the anesthesiology group annually.

Straightforward recommendations to reduce harm exist—avoid desflurane and nitrous oxide, use lower fresh gas flows (FGF), and utilize total intravenous anesthesia and regional techniques.^20–23^ However, despite the publication of these recommendations, systematic changes to clinical practice remain challenging.

Global warming potentials (GWPs) of GHGs are defined over 100 years (GWP100) based on their impact with carbon dioxide as the reference (defined as GWP = 1). Using published calculations^20,24^ for converting volatile anesthetic concentrations to carbon dioxide equivalents (CO_2_e), we derived the average CO_2_e (kg/min) for every anesthetic administered at our pediatric ASC between January 2018 and July 2022.

Practice constraints included removing desflurane from the pharmacy formulary, reducing the anesthesia machine’s default FGF (8–2L/min), and unplugging nitrous oxide hoses. There was consistent departmental education. We implemented clinical decision support tools alerting providers to decrease flows if FGF and volatile concentrations were above a set threshold. Finally, real-time emissions data for each anesthetic performed in the operating rooms meant we could discover cases and which anesthesia team members had the highest and lowest emissions to further target interventions.

Inhaled anesthetic GHG emissions decreased 87% over four and a half years through three main phases of interventions, each with SCV. Baseline emissions were 0.42 kg CO_2_e/min (from January 1, 2018, to November 30, 2019). Phase 1 interventions included starting educational efforts and lowering the default anesthesia machine FGF in the operating rooms (November 2019), which decreased emissions by almost half to 0.23 kg CO_2_e/min. Phase 2 interventions included an EMR alert for FGF (May 2021) and ongoing education, with emissions averaging 0.15 kg CO_2_e/min. Finally, another SCV was seen after the start of Project Saving our Planet by Reducing Carbon Emissions (December 2021), followed by phase 3 interventions, including monthly email updates and unplugging the nitrous oxide hoses (January 2022), resulting in a new stable level of emissions at 0.05 kg CO_2_e/min (Fig. 5, modified figure^25^).

## DISCUSSION

### Summary

Our ASC exemplifies an LHS in clinical practice on a microsystem level. Over the past 7 years, our data infrastructure paved the way for continuous quality and safety improvement, allowing quick adaptations to care plans that improve patient care and outcomes. Before our current data infrastructure, it would take months to years to collect data to analyze and determine if our processes were leading to improvement. Now, the same ideas can manifest and provide results in real time. In addition, our perioperative team members’ progressive growth mindset and culture allow for integrating ongoing practice changes with minimal hurdles. Best practice measures will change as health technology advances equipment and procedural techniques. Integrating these changes will be pivotal to transforming healthcare for future generations. Through a series of simultaneous, diverse QI projects, our perioperative team has analyzed and interpreted real-time data over several years to change our perioperative practice. With each project, we catapulted our ability to see standardized practice from a different perspective than our predecessors. We have optimized urology surgical block time use, worked to maintain adequate analgesia while further reducing perioperative opioid use for T&A, created a center-wide pediatric ERP that has further reduced postoperative pain, evaluated for racial disparities, and lastly, improved safety by tracking and reducing our facility’s GHG emissions. From a financial perspective, our single-center ASC does not provide financial incentives to reward high-value care. However, our cultural mindset promotes performance transparency, which allows the entire team to benefit and learn from everyone’s success.

This QI initiative has several notable strengths: (1) Using easily accessible, continuously updated real-world data promptly allowed the improvement team to complete multiple PDSA improvement cycles rapidly and simultaneously. For example, leaders published individual anesthesia team members’ average carbon footprint for our environmental project on a shared dashboard and highlighted in monthly email messages as part of Project Saving our Planet by Reducing Carbon Emissions, increasing motivation to make further reductions. A funnel plot displays data ordered from low to high performers. One of the highest emitting CRNA (A) decreased emissions from 0.92 to 0.45, then 0.32, and finally 0.08 kg CO_2_e/min by the third stage of interventions (Fig. 6). A below-average emitting CRNA (B) led the group with reductions from 0.31 at baseline to 0.23, 0.1, and finally, 0.03 kg CO_2_e/min. (2) For the pediatric ERP project, the team was able to build upon a strong foundation of previously published opioid-sparing anesthetic techniques^13,26–28^ and easily focus on a center-wide versus procedure-specific ERP approach due to the universal opioid-sparing anesthetic practice and ease of access to the center-wide outcome, process, and balancing measures. The ability to successfully launch a center-wide ERP likely represents the culmination of our progressive efforts to use all aspects of the LHS in our ambulatory practice. (3) The entire QI effort was significantly facilitated by the stable and sustained continuous learning culture present.

#### Limitations

Only the urology surgical service participated in our efficiency project. There currently needs to be more cases for the equity project to determine if our intervention will lead to SCV.

The findings from our LHS implementation are a single center’s experience and are not easily generalizable. However, many surgery centers have a similar technological foundation—an EMR, capturing each patient’s episode of care. Unfortunately, leadership, mindset, ease of data access, total data transparency, and a culture embracing and expecting *continuous* change is harder to replicate and sustain.

#### Conclusions

BCSC has transformed into an LHS in clinical practice due to our ability to make changes in our system and assess in real time if those changes are effective. By monitoring our electronic health record data, we can determine if changes to our system are real improvements. In doing so, what we used to consider as one-off “QI projects” are now a series of continuous improvement cycles. With strategic goals of quantitatively improving efficiency, effectiveness, efficacy, equity, and environmental safety, our patient-centered care has drastically improved.

## ACKNOWLEDGMENTS

We thank Dr. Paul Sharek for his editorial assistance.

## Supplementary Material


