# Supplementary figures and images for: Transforming into a Learning Health System: A Quality Improvement Initiative
Source: Pediatr Qual Saf. 2024 May 9;9(3):e724. doi: 10.1097/pq9.0000000000000724 (PMC11093568; doi:10.1097/pq9.0000000000000724)

Effectiveness: Supplemental Figure 1

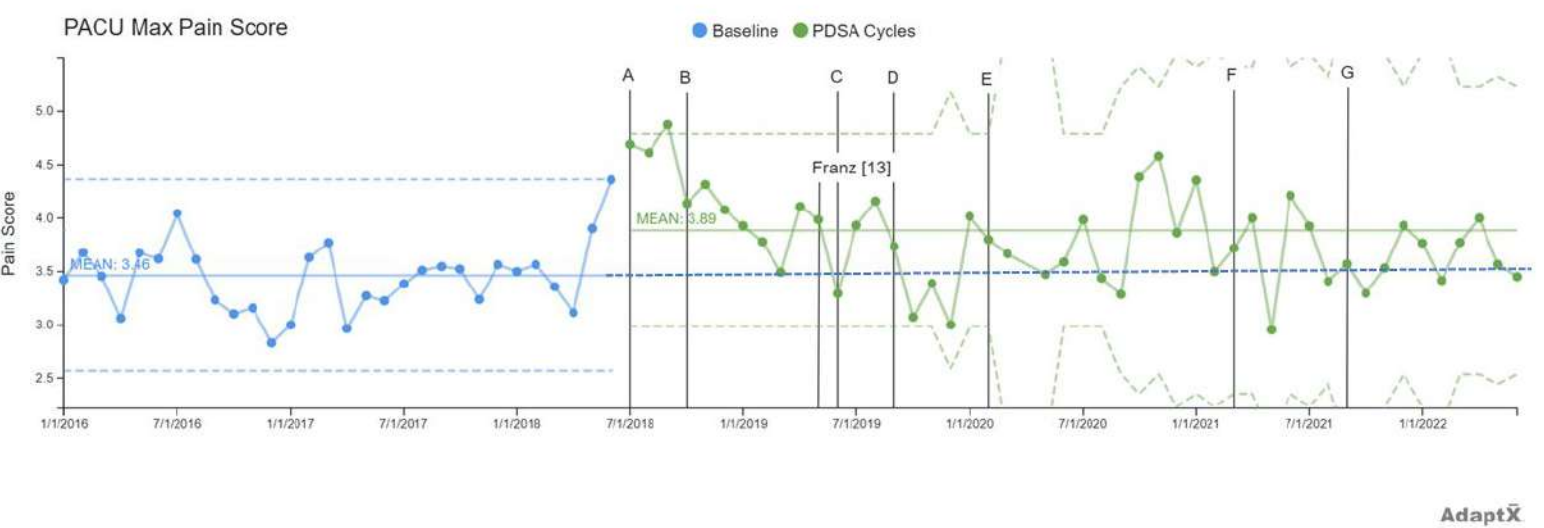

Supplement: Supplementary file 1 [file pqs-9-e724-s001.pdf]

Effectiveness: Supplemental Figure 2

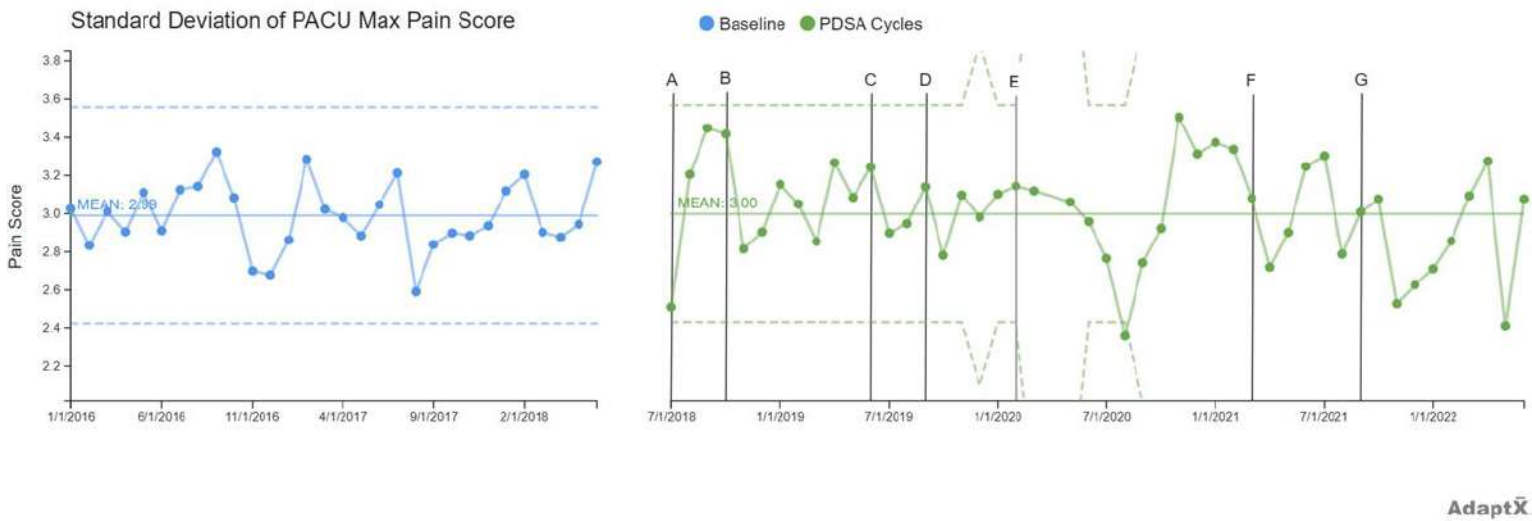

Supplement: Supplementary file 2 [file pqs-9-e724-s002.pdf]

Efficacy: Supplemental Figure 3

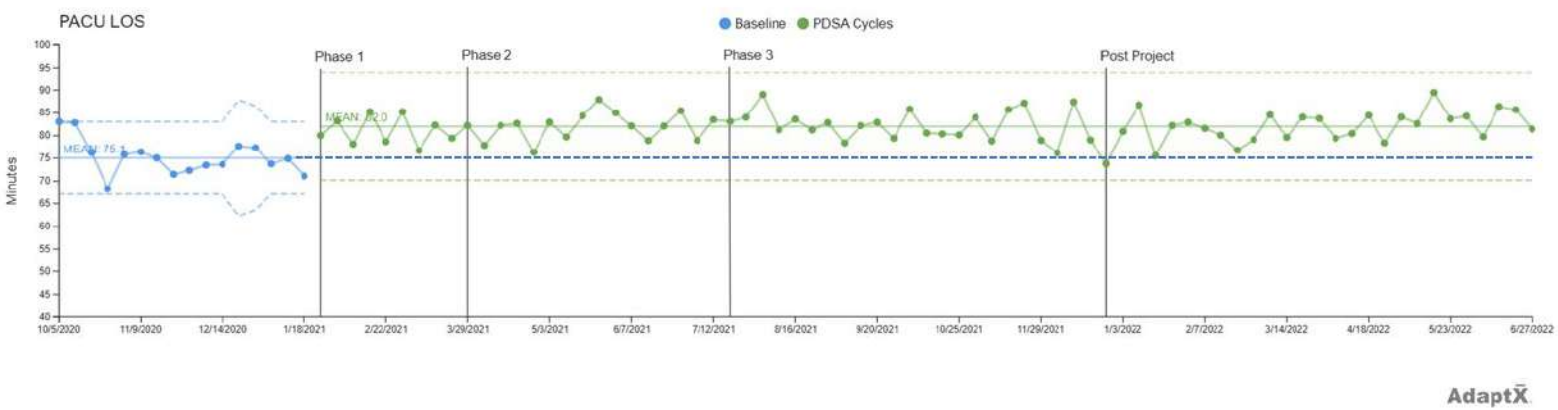

Supplement: Supplementary file 3 [file pqs-9-e724-s003.pdf]

Efficacy: Supplemental Figure 4

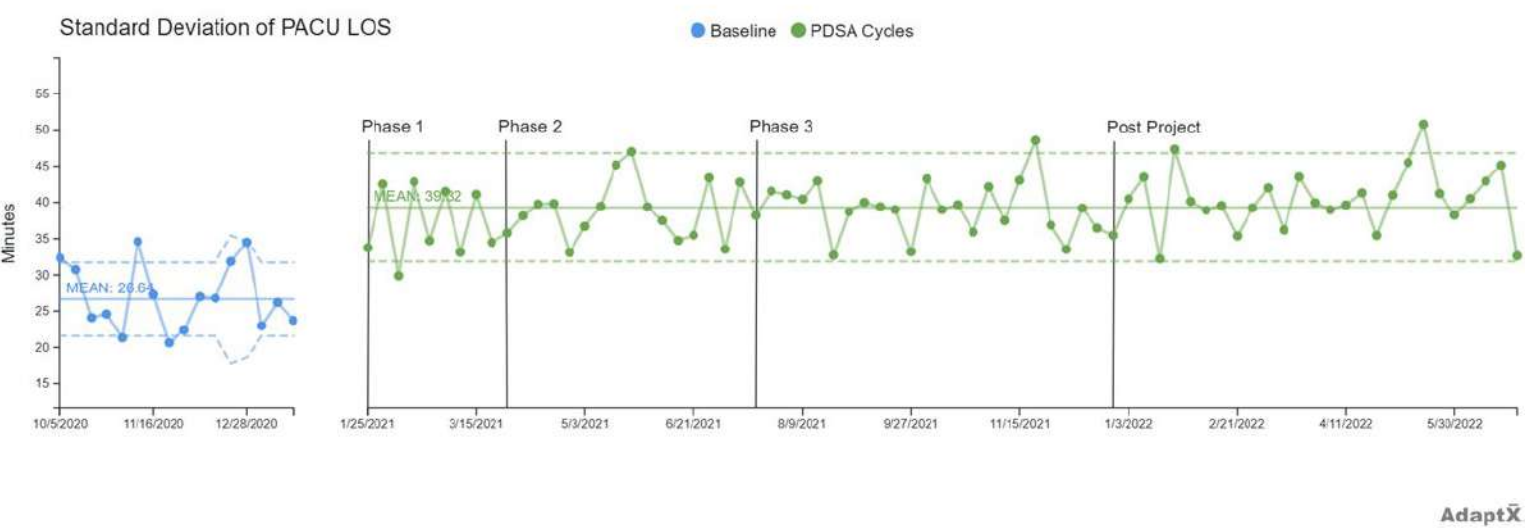

Supplement: Supplementary file 4 [file pqs-9-e724-s004.pdf]

Efficacy: Supplemental Figure 5

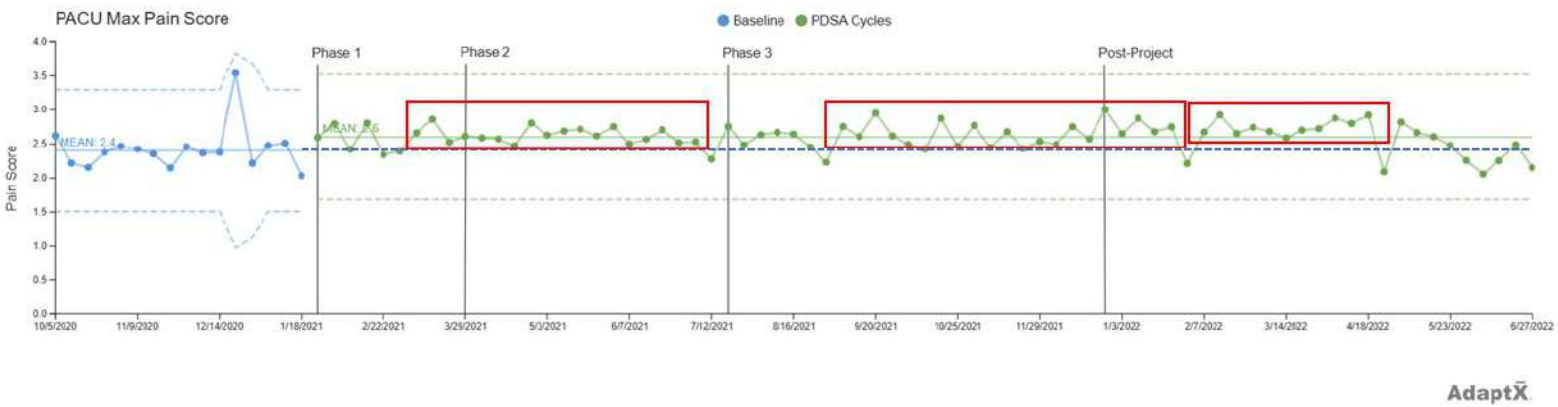

Supplement: Supplementary file 5 [file pqs-9-e724-s005.pdf]

Efficacy: Supplemental Figure 6

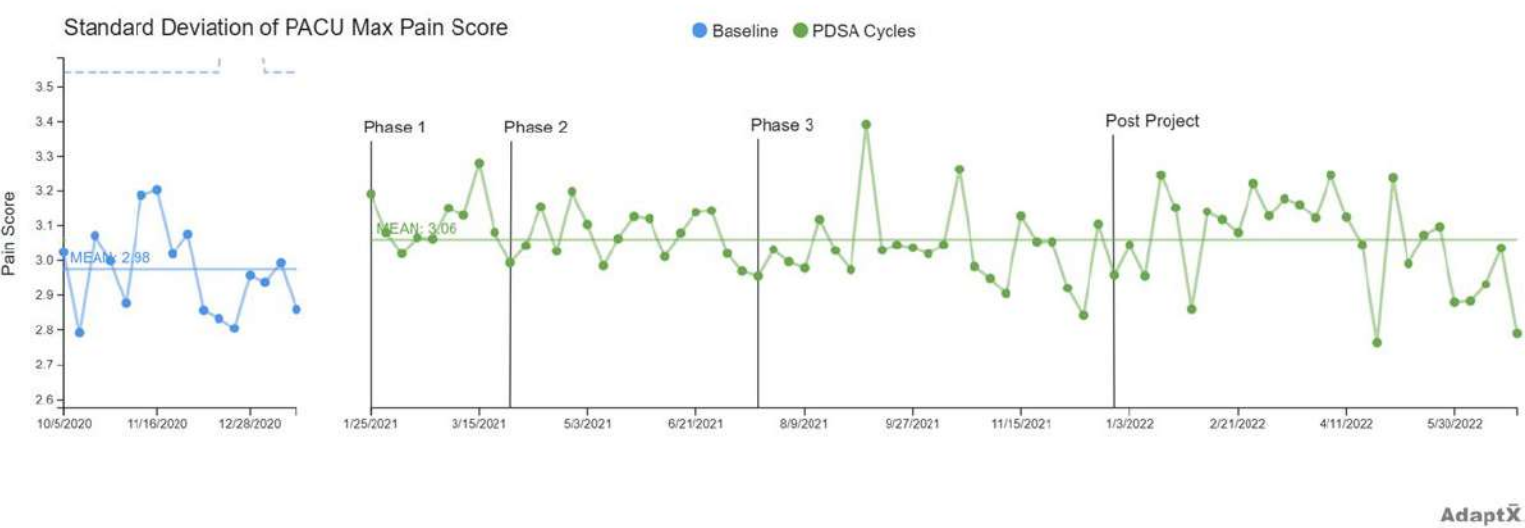

Supplement: Supplementary file 6 [file pqs-9-e724-s006.pdf]

Efficacy: Supplemental Figure 7

PACU Rescue IV Opioids Rate

● Baseline ● PDSA Cycles ● PDSA Cycles

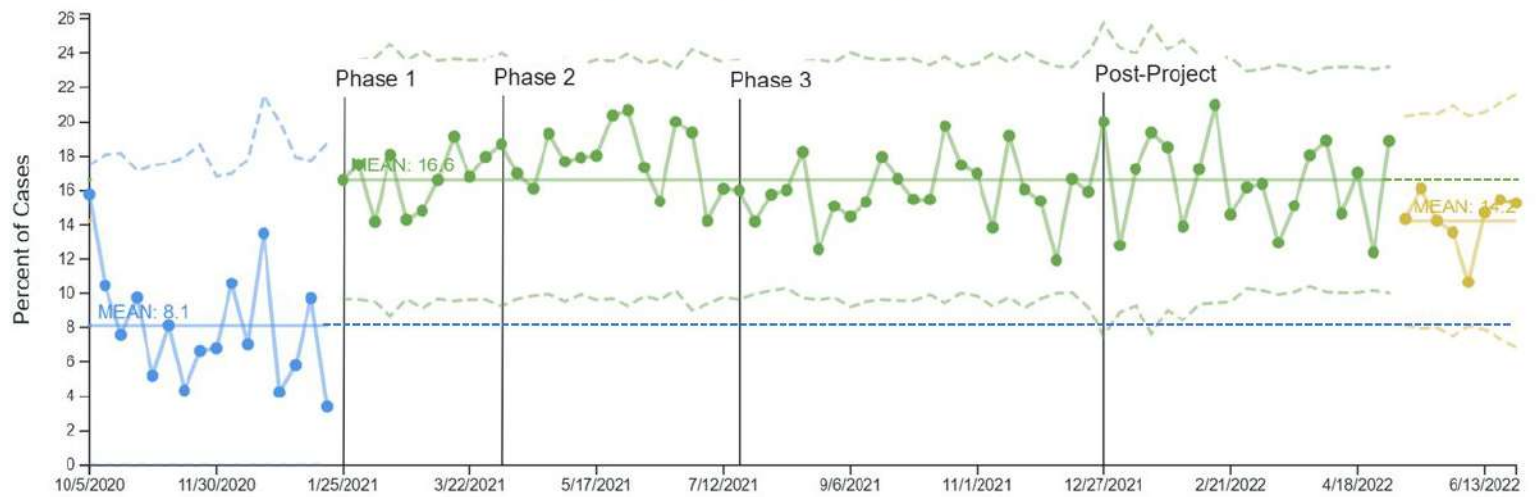

Supplement: Supplementary file 7 [file pqs-9-e724-s007.pdf]

Efficacy: Supplemental Figure 8

Standard Deviation of Post Op Opioid Prescription Dose Count

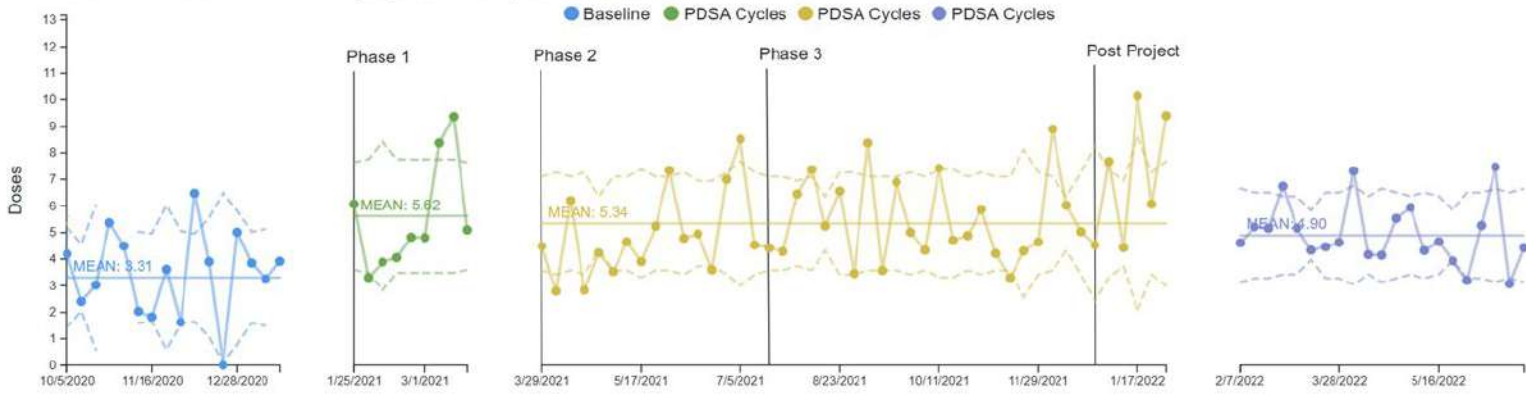

Supplement: Supplementary file 8 [file pqs-9-e724-s008.pdf]
